# The odyssee from surveillance to the detection of pancreatic cancer, total pancreatectomy, and its impact on life. insights from a p16-Leiden pathogenic variant carrier

**DOI:** 10.1007/s10689-024-00385-0

**Published:** 2024-04-25

**Authors:** Amarensia (Marit) Spruitenburg, Hans FA Vasen

**Affiliations:** 1 Department of Gastroenterology & Hepatology, Leiden University Medical Centre, Leiden, The Netherlands; 2 Familial Cancer & Department of Gastroenterology & Hepatology, Leiden University Medical Centre, Leiden, The Netherlands

**Keywords:** Hereditary pancreatic cancer, CDKN2A pathogenic variant, Surveillance, Early detection, Total pancreatectomy, Patient’s perspective

I will begin my story in March 2001. That’s when pancreatic cancer was diagnosed in my sister. She passed away a month later, leaving us bewildered and sorrowful. She was 49 years old. Shortly after her death, my brother suggested that we contact the Clinical Genetics Center. Our mother had died young, and her sister had also succumbed to pancreatic cancer. My brother had heard from someone that there are families in this region with a hereditary predisposition for melanomas and pancreatic cancer. Both my sister and I had experienced melanomas (Fig 1.)

**Fig. 1:**
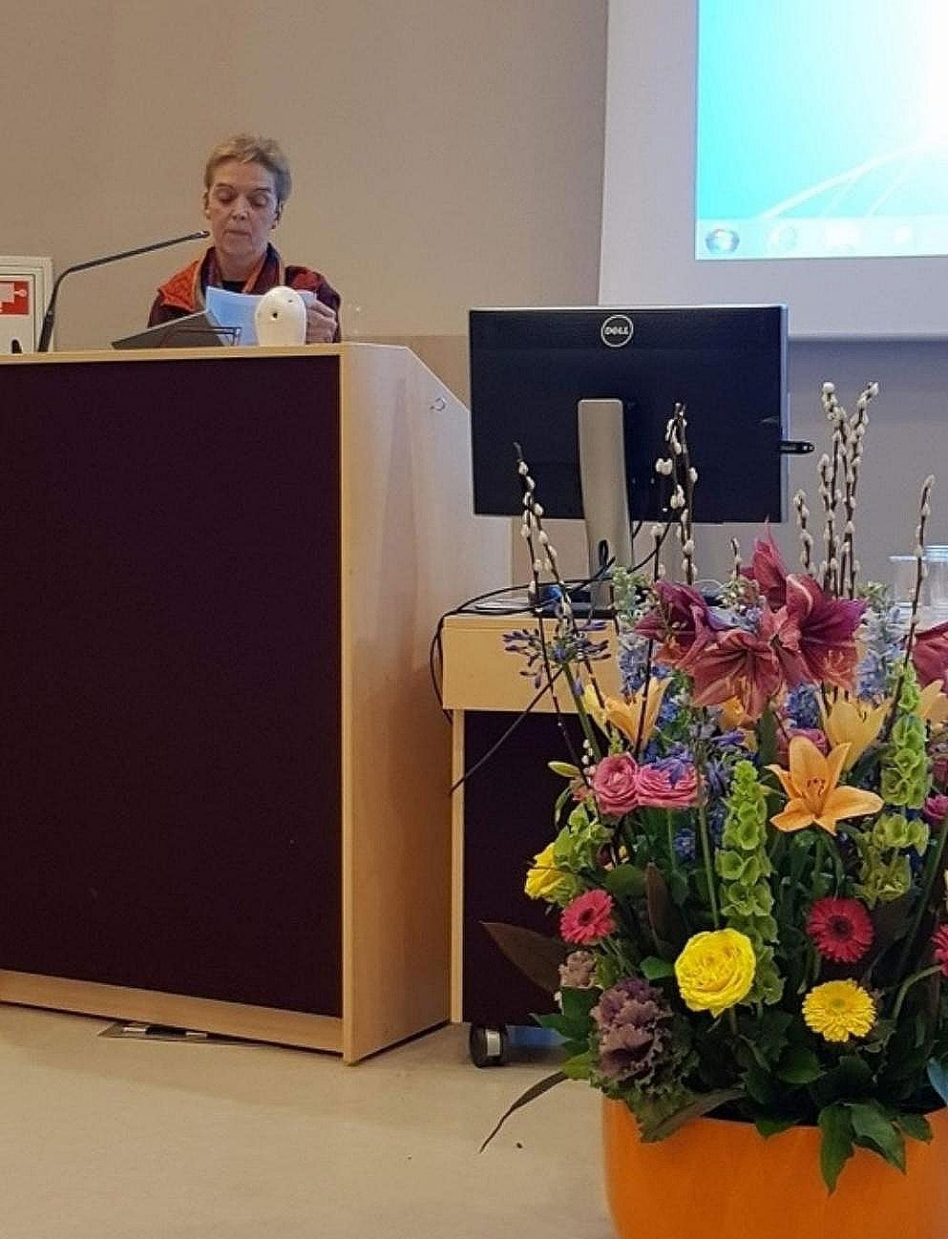
Amarensia Spruitenburg at the patient education event in Leiden

The ball started rolling, and my brother and I were found to have the p16-Leiden mutation. I was 46 years old at the time and immediately joined Dr. V.‘s research. Every year, I underwent an MRI scan, discussing the results with Dr. V. I wasn’t overly concerned because I couldn’t imagine more children in our family getting pancreatic cancer. The chance seemed small. The conversations with Dr. V. were interesting and enjoyable. I liked going there. My brother, being younger, joined the study years later. In 2009, he had his first MRI scan, and it confirmed pancreatic cancer. He was 49 years old. He underwent surgery performed by Dr. B., and I accompanied him to the appointments. I paid close attention, knowing I could be next. My brother lived for almost three more years, and they were good years.

After his death in 2012, I thought I might escape the fate. Two children from a family seemed enough. In late 2016, I had another MRI scan, and in early 2017, I had an appointment with Dr. V. for the results. I was very sick with the flu and canceled the appointment. In the afternoon, Dr. V. called and informed me that he had seen a tiny spot in the head of the pancreas on the scan. He wanted to investigate further and scheduled an endoscopic ultrasound. I wasn’t very panicked. I couldn’t imagine it being anything other than pancreatic cancer, but I thought we were catching it very early. I discussed the situation extensively with my partner, some family members, and good friends. I continued working and asked my general practitioner for sleeping pills to prevent anxious thoughts at night.

In early February 2017, I underwent the endoscopic ultrasound. A tube with an ultrasound device was inserted, and I didn’t notice it due to the sedation I received. Afterward, I was told that a small spot was observed. Tissue was taken for examination, not from the spot Dr. V. had pointed out, but from the middle of the pancreas.

During the discussion of the endoscopic ultrasound with Dr. V., he mentioned the presence of at least one small malignant tumor, but he wondered if there might be more small tumors. He had seen another spot on the tail of the pancreas when reviewing the scan carefully. Given my family history, he proposed removing the entire pancreas. Initially, I didn’t even know it was possible to live without a pancreas, but if it was, I was ready to have the whole thing removed. Besides the pancreas, they would also remove the gallbladder, duodenum, and spleen. We discussed the “quality of life” because, without a pancreas, you immediately develop diabetes type 1 and need to take digestive pills for life. I felt I could handle that. I’m naturally disciplined, not a big eater, and have never smoked or drank alcohol.

After the appointment with Dr. V., I went to a bookstore and bought an atlas of the human body. It was high time to study what it all looked like inside, what would be removed, its function, and how the new “pipes” would be connected. I did a lot of online research. There wasn’t much information in Dutch about “living without a pancreas”, but there was an American site where people who had their pancreas removed shared their experiences.

I found it interesting. It was just unfortunate that it was about me. I felt like I was in the wrong movie. After that, I entered a sort of whirlwind. Appointments followed with Dr. VM, one of the surgeons who would operate on me. I visited the diabetes nurse who told me about my future life with diabetes type 1. I received a kit with various items I would need, and she mentioned that I would have to carry that kit with me for the rest of my life. I still carry it with me now. I can show it to interested people later.

In the last week before the operation, I couldn’t concentrate well. I called in sick at work. In the back of my mind, I had a scenario that everything could go completely wrong. That’s why I cleared and threw away personal belongings. I checked if my will was in order. I even joined the Association for Voluntary Euthanasia. During that week, I cycled a lot. The weather was exceptionally sunny with high temperatures.

On March 21, it was time. I vaguely remember being taken to the operating room, where a complete team was ready, each introducing themselves separately. I knew Dr. VM. If I remember correctly, he told me that Dr. B. was on the way. I knew that the first step was to check for any metastases. If found, there would be no operation. I trusted it completely. I was going to be operated on in a top hospital by top surgeons. And if it went wrong, I had still reached the age of 61 with a very faulty pancreas.

The next thing I remember is someone mentioning my name a few times, and I asked what time it was. It was the next day, 6 o’clock in the morning. I had been operated on. YES! My partner had been there the previous evening. I didn’t notice anything. One of the nurses had told him that I had mumbled the word ‘life.’ I can imagine that because I knew I had a good chance of at least temporarily surviving if the operation went through.

I spent two weeks in the hospital. In the first week, I slept a lot. Almost every day, some tube or another could be removed. I was allowed to, or rather, I had to sit on a chair and take short walks quite soon. Eating wasn’t working, and a tube was inserted for liquid food directly into the vein. I was impressed by the expertise, dedication, patience, and kindness of all the hospital staff I encountered. A new world opened up for me.

Dr. B. came to tell me that ultimately, only one real tumor of 9 mm was found in the pancreas. Later, we discussed the structure of the pancreas, which turned out to be full of ‘PanIN.’ If I understood correctly, that’s a kind of precancerous stage.

As I started feeling better, it became somewhat cozy with my fellow patients. We often felt unwell, but between the bouts, we also had fun. The visiting hours were enjoyable, and I received flowers, cards, and gifts. All the attention did me good.

At some point, I had to monitor my blood sugar level and inject insulin myself. It wasn’t so simple, and I was glad the nurses helped me.

Once I started getting enough nutrition, I recovered quickly. It was wonderful to be able to use the toilet, shower, and take a walk around the ward. I could climb stairs, and I occasionally practiced on the exercise bike. I enjoyed the food, but before I could truly savor it, the doctors started talking about going home. A phrase I occasionally heard was that the hospital didn’t keep lodgers.

On a Friday afternoon, before my last weekend in the hospital, Dr. V. visited, and I remember that visit as inspiring and pleasant. I was grateful to him because, through the screening all those years, I was likely caught in time, and because he didn’t trust a tiny dot on the MRI scan. I think the same day, Dr. B. also came to say that chemotherapy was recommended in my situation. Adjuvant chemotherapy. Supplementary to the surgery. I wasn’t looking forward to it, but I didn’t find it a big problem. I felt I could take on the whole world.

On the evening of April 3, I went home. I could have left earlier that day, but at my request, I got to enjoy the delicious dinner one last time. The weather was beautiful again that day, and as I sat in the car, all the colors seemed to have become brighter along the way. The clear blue sky, the green grass, the blooming trees. I felt incredibly happy. My partner had hung the flag. The guest bed mattress lay on the living room floor. Family and friends came by with flowers. I felt like I was on top of the world. I was alive.

Rehabilitating at home had its ups and downs. Two steps forward, one step back. Eating and drinking remained a problem. I lost kilograms. Walking and cycling in short distances worked. I could quickly drive a car again. In mid-May, I even went swimming. I live by a canal, and as soon as the temperature allows, I go swimming with my neighbors. They stayed close to me during swimming all summer because they were afraid something might go wrong. I felt my stomach for months. I wondered if it would pass. It did. Now I only feel my stomach when I eat too much, too fast, or too fatty.

At the end of May, I started chemotherapy and quickly became very sick from it. The dose was reduced, but it didn’t help. At one point, I spent a whole day in the hospital because I felt so sick. After that, in consultation with the chemo doctor, I stopped.

In late August, I had a small bleeding in the new connection between the stomach and the small intestine. I spent three days in the hospital and fortunately recovered quickly. However, I had lost weight again. I weighed 56 kg, while before the operation, I was 68 kg. Now I’m about 61 kg. To achieve that, I had to eat a lot. For example, for breakfast, I have oatmeal with cream, all without sugar because of the blood sugar level.

From early September, things got better. At first, I was afraid I would never leave the village again, then I was afraid I would never leave the Netherlands again, but in September, I drove to Germany by myself. I was a bit scared to be so far from the hospital, but everything went fine. Walking was going well, eating was going well.

So, I could still go on vacation. I was very happy about that. Last spring [2018], I even hiked in the mountains for a day. There was still a lot of snow on the peaks, and that day, actually for the first time, I cried. Out of pure happiness. I had never thought I would see mountains, let alone hike in them.

Last August, we cycled through the Netherlands for almost two weeks. For clarity: on regular bikes, not e-bikes. I had received various tips from the hospital dietitian and diabetes nurse because it wasn’t so easy to do something like that with diabetes. Eat a lot, inject less insulin. I got used to it.

I’m doing well. I should mention that I no longer work. I have brought forward part of my pension, and I will receive less money from the age of 67, but I’ll have to wait and see if I reach that age. Household chores, groceries, cooking, it takes much more energy than before. I lead a relatively quiet life. I often call it a life in the second gear, but I am very satisfied with it and enjoy it. I like to read, occasionally visit a museum, go to the movies, have coffee with my friends. Sometimes I say to my partner that these might be the best years of my life. I don’t have to prove myself anymore, I don’t have to perform anymore. I don’t have to do anything anymore. Of course, my situation is entirely different from someone twenty years younger, with a job and three children. Then it becomes a different, more complicated story.

I feel like a privileged person to be here today. Together with the doctors who, in my opinion, saved my life. I also realize very well that this is only possible because my cradle stood in a wealthy country. Otherwise, I would have been hopeless. Thank you for listening to my story. I wish you all the best.

## Data Availability

No datasets were generated or analysed during the current study.

